# Editorial: Exploring the complexities of the human nervous system through advanced brain imaging and stimulation

**DOI:** 10.3389/fnhum.2026.1936559

**Published:** 2026-08-14

**Authors:** Jiajun Yu, Xiaogang Xu, Pei Shang, Mingqin Zhu, Song Qiao

**Affiliations:** 1 School of Pharmaceutical Sciences, Wenzhou Medical University, Wenzhou, China; 2 Clinical Research Institute, Zhejiang Provincial People's Hospital (Affiliated People's Hospital), Hangzhou Medical College, Hangzhou, China; 3 Department of Neurology, Mayo Clinic, Rochester, MN, United States; 4 Department of Neurology, Neuroscience Center, The First Hospital of Jilin University, Changchun, China; 5 Department of Neurology, Zhejiang Hospital, Hangzhou, China

**Keywords:** acupuncture, brain imaging, cerebral small vessel disease, cerebrovascular disease, epilepsy, functional ultrasound, moxibustion, neuromodulation

The human nervous system is characterized by remarkable structural, vascular, metabolic, and functional complexity. Across cerebrovascular disease, epilepsy, neurodevelopmental conditions, neuro-oncological disorders, and acquired brain injury, this complexity contributes to substantial heterogeneity in clinical presentation, prognosis, and treatment response (Pantoni, 2010). Conventional clinical evaluation and routine investigations often confirm that neurological dysfunction is present, yet, frequently fail to explain why patients with similar diagnoses differ in symptom burden, disease course, or recovery potential. Structural lesions, vascular pathology, metabolic disturbance, and network-level disruption may coexist, but are not always discernible from bedside assessment alone. Equally important, clinicians often lack objective measures for determining whether an intervention has altered brain function rather than symptoms only. Advanced brain imaging, therefore, plays an increasingly important role not only in identifying lesions, but also in clarifying disease mechanisms, recognizing biological heterogeneity, and supporting more objective evaluation of functional change.

Brain imaging has moved beyond its traditional function of detecting focal lesions. Magnetic resonance imaging, together with functional imaging (Fox and Raichle, 2007), diffusion imaging (Le Bihan, 2003), and perfusion or metabolic imaging (Detre et al., 2012), now allows abnormalities to be examined at structural, vascular, metabolic, and network levels within the same patient. Rather than simply documenting where injury is visible, these approaches increasingly help explain how disease is organized, why subgroups differ, which patients may follow a less favorable course, and whether treatment is associated with measurable change in brain activity, connectivity, microstructure, or hemodynamics. In this sense, imaging is shifting from descriptive lesion reporting toward mechanism-oriented interpretation, prognostic refinement, and intervention evaluation. This evolving role is particularly relevant for disorders in which routine scans appear modest but clinical outcomes vary widely.

The articles collected in this Research Topic share a common translational premise: nervous system disorders cannot be fully understood through a single anatomical or clinical lens. Imaging markers can reveal hidden dimensions of disease burden, while stimulation and other modulatory approaches raise the complementary question of whether measurable brain states can be influenced in a clinically meaningful direction. The purpose of this Editorial is therefore not to present a single disease-centered narrative, but to highlight how diverse imaging modalities and intervention paradigms converge on a broader problem in human neuroscience: how to connect brain structure, vascular physiology, metabolism, connectivity, and behavior in ways that are informative for prognosis, stratification, and treatment evaluation.

In this Research Topic, a total of 12 articles were published that examine nervous system complexity through advanced brain imaging and related modulatory interventions. Several contributions focused on cerebrovascular and small-vessel disease. Ding et al. reported that deep tiny flow voids on high-resolution MRI were associated with milder deficits, smaller infarcts, and more favorable outcomes in acute middle cerebral artery atherosclerotic occlusion. Wang et al. showed that different white matter hyperintensity distribution patterns differ in vascular risk burden, gray matter volume, microstructural injury, and cerebral blood flow. In two complementary CSVD studies, Lan, Lei, Wang et al.; Lan, Lei, Xu, et al. linked deep medullary vein dysfunction to cerebral microbleeds and interstitial fluid changes, and demonstrated that basal ganglia enlarged perivascular spaces were associated with increased white matter free-water content through venous dysfunction. Together, these studies indicate that multimodal MRI can refine prognostic assessment and reveal vascular, microstructural, and interstitial-fluid abnormalities that are not captured by conventional imaging alone (Wardlaw et al., 2013).

Other articles extended the theme of imaging-based characterization to epilepsy, neurodevelopment, oncology, and toxic brain injury. Sun et al. reported that visual stimulation reduced epilepsy susceptibility in a neonatal hypoglycemic brain injury rat model with MRI-defined occipital injury. Liu et al. used functional ultrasound to characterize cerebral blood volume changes and network remodeling during acute pilocarpine-induced seizures in mice, in line with preclinical evidence that functional ultrasound can monitor brain hemodynamics at high spatiotemporal resolution (Macé et al., 2018). Huang et al. linked prenatal maternal depression and anxiety to altered limbic white matter microstructure in 8-year-old children, consistent with developmental neuroimaging work showing how early-life environments shape brain structure (Gilmore et al., 2018). Mao et al. described an MRI sign associated with brain invasion in meningiomas and metastases, and Li T. et al. reported MRI features supporting early recognition of occupational toxic encephalopathy. These studies emphasize that imaging can be useful across very different clinical settings when the central question is how brain pathology is organized, distributed, and linked to functional vulnerability.

A further set of studies evaluated stimulation and other modulatory interventions with imaging-based endpoints. Hu et al. used task-based fMRI to identify shared and acupoint-specific brain network responses to acupuncture (Hui et al., 2010). Zhang et al. reported cognitive improvement and altered functional connectivity after moxibustion in vascular dementia, whereas Li S. et al. synthesized randomized evidence that acupuncture was associated with improvements in selected MRS markers (e.g., NAA/Cr and Cho/Cr) and neurological outcomes in ischemic stroke. These contributions are especially relevant because they position imaging not only as a diagnostic tool, but also as a way to monitor how interventions may affect activation patterns, functional connectivity, or metabolic signatures. At the same time, they should be interpreted cautiously: imaging change does not by itself prove clinical efficacy, and intervention studies require rigorous design, replication, and clinically meaningful endpoints.

Collectively, the studies in this Research Topic highlight the value of advanced brain imaging as a bridge between disease characterization, mechanistic investigation, and objective evaluation of functional modulation and recovery. Advanced brain imaging makes structural, vascular, metabolic, and network abnormalities measurable, while stimulation and other modulatory interventions provide opportunities to evaluate and influence brain function and recovery. The Research Topic is not dominated by invasive brain stimulation technologies; rather, its strength lies in showing how diverse imaging approaches can clarify heterogeneity across vascular, epileptic, developmental, oncological, and toxic brain disorders, while a smaller but important subset of articles demonstrates how non-invasive modulatory interventions—including visual stimulation, acupuncture, and moxibustion—can be assessed through changes in activation, connectivity, metabolism, or seizure susceptibility.

Several priorities emerge from this Research Topic. First, future work should move from isolated markers toward integrated models that combine structural, vascular, metabolic, and network-level information. Second, imaging biomarkers need stronger links to outcomes that matter to patients, including cognition, disability, seizure burden, recovery trajectory, and treatment response. Third, methodological harmonization will be essential: multicenter studies, standardized acquisition protocols, transparent preprocessing pipelines, and longer follow-up are needed to determine which imaging features are robust, reproducible, and clinically transferable. Finally, intervention studies should distinguish between biological plausibility, imaging responsiveness, and durable clinical benefit. This distinction is important because brain imaging can sensitively detect change, but such change must be connected to meaningful functional improvement before it can guide routine clinical decision-making.

Much remains to be established. Future work will require multicenter collaboration, larger samples, standardized imaging protocols, and longer follow-up to clarify the relationship between imaging markers and meaningful clinical benefit, and to verify the real-world efficacy of different modulatory interventions. Nevertheless, the articles assembled here provide a coherent illustration of how advanced brain imaging and targeted intervention research can jointly address the complexities of the human nervous system. By bringing together vascular imaging, diffusion and functional MRI, functional ultrasound, MRS, and non-invasive modulatory approaches, this Research Topic underscores a central message: understanding nervous system complexity requires tools that can measure brain organization at multiple levels and evaluate whether interventions alter that organization in ways that are biologically interpretable and clinically relevant.
